# Editorial: Perinatal mood symptoms and postpartum maternal functioning: Describing the evidence related to effective and ineffective interventions

**DOI:** 10.3389/fpsyt.2022.975078

**Published:** 2022-07-28

**Authors:** Jennifer L. Barkin

**Affiliations:** Mercer University School of Medicine, Macon, GA, United States

**Keywords:** postpartum functioning, perinatal mental health, maternal functioning, Barkin Index of Maternal Functioning, climate anxiety

The goal of this special issue entitled, “*Perinatal mood symptoms and postpartum maternal functioning: Describing the evidence related to effective and ineffective interventions*,” was to highlight interventions that have been evaluated in peer-reviewed research in regards to their efficacy toward improving perinatal mood symptoms and/or postpartum maternal functioning. While there is a comparatively rich body of research surrounding perinatal depression and anxiety, postpartum maternal functioning has exacted less attention, to date (1–3). There are several reasons for this including: (1) an early and almost exclusive focus on postpartum depression (PPD) as the primary mental health concern of interest in new mothers (4), (2) a lack of current, quick, and accurate tools to assess postpartum functional status (2), (3) inadequate attention paid to mothers and their needs post childbirth (5, 6), and a predominant focus on infant health and the clinical outcomes of the pregnancy (5). However, over the past 10 years, assessment of maternal functioning has made its way into the conversation, and also into domestic (7–10), international (11–13), and industry-sponsored studies (14, 15). Though the evidence base is still growing, we do know that some interventions seem to improve maternal functioning. For example, women who participated in the Visiting Moms^®^ program in Waltham, Massachusetts (*n* = 149), scored, on average, 16 points higher on the Barkin Index of Maternal Functioning (BIMF) (1) at program completion (relative to program intake) (4). This intervention included weekly home visits from trained volunteers through the baby's first year of life and corroborates the knowledge base indicating that social support is a protective factor (4). Postpartum patient education *via* the Skills Training Approach (STA) also appeared efficacious at improving maternal functioning—across all domains—in a small randomized controlled (*n* = 68) trial of Iranian women (11). In fact, the group of women who received maternal skills training shortly after childbirth had an average BIMF score of 95.8 vs. 70.3 in the control group (11). Stated differently, the women who received postpartum education were functioning, on average, 25 points higher than those who received usual care, making the case for more education-centered approaches to functional improvement. Clinical interventions such as the Hennepin Healthcare Mother-Baby Day Hospital, where perinatal women with severe to moderate psychiatric illness receive trauma-informed group-based therapy and psychiatric care, have shown great promise and significant improvements in depression, anxiety, and maternal functioning (16). Intensive Outpatient Programs (I.O.P.s) have likewise reported success in achieving functional improvement in postpartum women (17).

Through this special issue, both promising interventions and risk/protective factors associated with perinatal mental health are explored. For example, Deif et al. examine the complex role of breastfeeding in relation to maternal mood (18), *via* a literature review focused on Dysphoric Milk Ejection Reflex (D-MER). Likewise, Iodice et al. consider the role of Oxytocin, concluding that the hormone may play a protective role in the development of perinatal depression. In a hygiene-focused study, Jiang et al. explored the association between caregiver hand washing practice and postpartum mental health; results from this cross-sectional study implicate suboptimal hand washing practice as a risk factor for maternal depression, anxiety, and stress. In terms of new behavioral health interventions, Flynn et al., Monteiro et al., and Peifer et al. each report compelling results and innovative programming. The special issue is nicely rounded out with an opinion piece by Albanese et al. who call for more patient-centered research and interventions for new mothers, whose *mental* health needs have been historically minimized (9).

While we continue to think about ways to support pregnant and postpartum women toward holistic mental health and optimal daily functioning, there is a looming threat (risk factor) on the horizon and its name is the climate crisis (5). In fact, the World Bank recently issued a report estimating that more then 200 million people are likely to be displaced due to climate change/extreme weather events (EWEs) over the next 30 years (19). We also know that women and children are vulnerable subgroups and are disproportionately affected (20). As the climate crisis intensifies, more pregnant and postpartum women will be impacted economically, socially, mentally, and physically. Organizations that assist new mothers, such as Postpartum Support International (PSI) (21), should strongly consider incorporation of climate change effects into their programming. Healthcare providers serving the perinatal population will need to consider environmental factors, including extreme heat, when assessing their patients' mental (and physical) wellness.

## Author contributions

The author confirms being the sole contributor of this work and has approved it for publication.

## Conflict of interest

The author declares that the research was conducted in the absence of any commercial or financial relationships that could be construed as a potential conflict of interest.

## Publisher's note

All claims expressed in this article are solely those of the authors and do not necessarily represent those of their affiliated organizations, or those of the publisher, the editors and the reviewers. Any product that may be evaluated in this article, or claim that may be made by its manufacturer, is not guaranteed or endorsed by the publisher.
